# Supplementary material for: Value, transparency, and inclusion: A values-based study of patient involvement in musculoskeletal research
Source: PLoS One. 2021 Dec 1;16(12):e0260617. doi: 10.1371/journal.pone.0260617 (PMC8635367; doi:10.1371/journal.pone.0260617)
Supplement: S1 Table — (PDF) [file pone.0260617.s001.pdf]

PPI Evaluation Project: Phone call questions and framework mapping

Value systems & descriptions (Gradinger 2013)	Revised question plan (telephone questions)
**Opening Questions**	1. Why did you join the patient involvement groups? ([PPI coordinator] to give a list of names and their groups to personalise this)<br>2. What do you like most about the group?<br><br>I will now ask some questions further to those given on your questionnaire. Some of the questions might seem similar but they are looking at slightly different aspects of the group. Please say if you do not wish to answer any questions or you wish to stop the interview at any point.<br><br>You should have received a letter about the interview being recorded. Are you happy to give permission for this?
**Normative value systems**	
**Empowerment**<br>Transfer of control, self-help, seeking to overcome discrimination and oppression.	3. How much do you feel involved in decisions made within the group?<br>4. Do you feel that you have received adequate training for your involvement?<br>5. A. What have you received that was helpful? / What additional training might you have wanted?
**Rights**<br>Refers to PI being of intrinsic value, about the fundamental human right to have a say.	6. How much do you feel that the researchers value your comments?
**Change / Action**<br>The idea of generating or translating knowledge into action in order to incite change.	7. What have you seen change as a result of your input on the research? What impacts have you noticed / seen happen following involvement with the PPI group?<br>8. Do you have further examples of this? Is there anything that you are proud of particularly?
**Accountability / Transparency**	9. How clear are you about the value of the research you have been involved in within the wider health context?

Public accountability and transparency about research and PI.	a. Do you feel that the research that you are involved in matters?
**Ethical values**<br>Ethical awareness in order to protect from harm.	10. Do you feel comfortable to share your views in the PPI meetings? (yes/ no)<br>a. What has helped you to feel able to do this? / What would help you feel able to do this?
**Process value systems**	
**Partnership / equality**<br>Sharing power and decisions in equal, reciprocal and collaborative PI processes.	11. How equal do you feel the relationship between researchers and group members is?<br>a. Could you give an example or tell me more about that?<br>b. How shared do you feel the decision making in the group is between group members and researchers?
**Respect / trust**<br>Respecting diversity, values, skills, knowledge, and experience in mutually beneficial PI processes.	12. How well do you feel that your personal skills and experiences are used within the group? Is there anything that might stop you participating fully?<br>13. What could be done to better support you?<br>14. Do you feel there is respect and trust between group members and researchers?
**Openness / honesty**<br>Processes and attitudes being open, honest, flexible and committed to PI.	15. Would you like more information about specific changes made following the group's input?<br>a. How would you like the details shared to you about what has changed?<br>b. How clear is it to you about what has changed because of the group's input?
**Independence**<br>Processes, facilitation and evaluation being independent.	16. Do you feel your priorities are heard and acted upon?<br>17. Do you feel the research discussed is important or relevant to you?
**Clarity**<br>Purpose, processes, communication and definition of PI being clear.	18. Do you feel clear about what is expected from you in the PPI group?
**Substantive value systems**	
**Effectiveness**<br>PI to actually have an effect on research and implementation.	19. At the end of the research projects that the group has been involved with, how much do you feel this patient group's involvement has impacts on how the results are shared, or whether any wider change in practice happens?
**Quality / relevance**<br>Increasing the quality, relevance and credibility of research through PI.	20. How do you feel that the group's involvement makes the research discussed more relevant to the general public?

**Validity / reliability**<br>Processing reliable, valid knowledge through PI.	21. Do you feel the PPI group impacts on the scientific quality of our research?<br><br>a. Could you expand on your answer?
**Representativeness / objectivity / generalizability**<br>Creating representative, objective, and generalisable knowledge through PI.	22. How well do you feel the PPI group represents the public being studied in the research studies?
**Evidence base**<br>Generating a substantial, consistent, comparable and replicable evidence base about PI.	23. What structure of meeting do you feel works best for the group?
**Ending questions**	24. Do you have anything else you would like to add?
