# Supplementary material for: Value, transparency, and inclusion: A values-based study of patient involvement in musculoskeletal research
Source: PLoS One. 2021 Dec 1;16(12):e0260617. doi: 10.1371/journal.pone.0260617 (PMC8635367; doi:10.1371/journal.pone.0260617)
Supplement: Attachment — Submitted filename: Response to reviewers.docx [file pone.0260617.s002.docx]

PLOS ONE Editorial Team

Carlyle House

Carlyle Road

Cambridge

CB4 3DN

16^th^ June 2021

Dear Sara Rubinelli and Reviewers,

Thank you for taking time to review our manuscript. We very much appreciate the constructive feedback. We have responded to each point raised in the table below. We hope these changes meet with your approval and we look forward to your response.

Yours sincerely,

Katie Whale

| **Feedback** | **Response / action taken** |
| --- | --- |
| **Editor feedback** | |
| 1. Please ensure that your manuscript meets PLOS ONE's style requirements, including those for file naming. | The formatting and style requirements have been checked and the manuscript updated. |
| 2. Please modify the title to ensure that it is meeting PLOS’ guidelines | Thank you, we have checked the guidelines and have added in information about the study basis. Title has been changed to: ‘Value, transparency, and inclusion: A values-based study of patient involvement in musculoskeletal research’. |
| 3. We note that you have indicated that data from this study are available upon request. PLOS only allows data to be available upon request if there are legal or ethical restrictions on sharing data publicly  In your revised cover letter, please address the following prompts:  a) If there are ethical or legal restrictions on sharing a de-identified data set, please explain them in detail (e.g., data contain potentially sensitive information, data are owned by a third-party organization, etc.) and who has imposed them (e.g., an ethics committee). Please also provide contact information for a data access committee, ethics committee, or other institutional body to which data requests may be sent.  b) If there are no restrictions, please upload the minimal anonymized data set necessary to replicate your study findings as either Supporting Information files or to a stable, public repository and provide us with the relevant URLs, DOIs, or accession numbers. For a list of acceptable repositories | Participants provided their consent to share questionnaire data and this was approved by the research ethics committee.  Participants did not provide their consent to share interview data, and this data is sensitive and contains potentially identifiable information and so this presents and ethical restriction on data sharing.  We have added the following wording as a data access statement:  Anonymised survey data is stored on University of Bristol Research Data Storage Facility and can be accessed via the University of Bristol Research Data Repository. Interview data is sensitive and contains potentially identifiable data and so cannot be shared. Access to survey data is restricted with data only available to bona fide researchers after a Data Access Agreement has been signed by an institutional signatory. Requests for access to the data may be made to [data-bris@bristol.ac.uk](mailto:data-bris@bristol.ac.uk). – Lines 661-666 |
| 4. Please review your reference list to ensure that it is complete and correct. If you have cited papers that have been retracted, please include the rationale for doing so in the manuscript text, or remove these references and replace them with relevant current references. Any changes to the reference list should be mentioned in the rebuttal letter that accompanies your revised manuscript. If you need to cite a retracted article, indicate the article’s retracted status in the References list and also include a citation and full reference for the retraction notice. | References have been checked. No retracted papers have been included. |
| **Reviewer 1 feedback** | |
| This is a nice study exploring the views of researchers and patients who are involved in a 'partnership in research' group. It is original research using appropriate methods guided by a relevant theoretical framework. The standard of writing is generally good. | Thank you for this feedback. |
| Lines 77-79 “However, it is hard to evidence the full scope of PPI impact because the wide range of activities and levels of patient involvement used in research can make it hard to quantify[10, 11].” – Does evidence of impact have to be quantifiable? Perhaps rephrase? Also, avoid using the same terms twice in a sentence (‘hard’). | Phrasing has been changed to: *However, some have suggested it is challenging to objectively evidence the full scope of PPI impact because the wide range of activities and levels of patient involvement used in research can make it hard to evaluate*– lines 78-79 |
| Lines 86-87 “... researchers have a moral obligation to redress any power imbalance through PPI [15-17].” What power imbalance? In the development of services? In the research or consultation process? Please clarify. | Thank you, this sentence reflects the contents of references 15-17 and so we have revisited these and made some additions so that the wording is more specific. We have added words as highlighted: ‘…that researchers have a moral obligation to redress any power imbalance in research and healthcare decision making through PPI’. – Line 89 |
| Line 94 “patient involvement work” ? | PPI is used throughout as an acronym and defined on first use. |
| Lines 119-122 Missing words in this sentence? “We wanted to use a theoretical framework to develop our understanding of PPI once the PEP-R activities were well embedded into the Research Unit and the two specialised groups had been running for several years, [so we???] we carried out a study of the three PPI groups at the Unit 2019 - 2020.” | Thank you, our apologies, we have added the missing word as follows:  *“We wanted to use a theoretical framework to develop our understanding of PPI once the PEP-R activities were well embedded into the Research Unit and the two specialised groups had been running for several years, we therefore carried out a study of the three PEP-R groups at the Unit in 2019 – 2020.” ­*– Lines 123-126 |
| Line 168 “clinical academics who had attended PPI meetings during the previous three years were” – do you mean PEP-R meetings? In fact, you refer to both PPI and PEP-R groups from here on. It seems you are using them interchangeably? I found that confusing. It seemed to me that you are evaluating your PEP-R group which is an example of PPI. | Thank you. We see now that this is very confusing, we have changed this to PEP-R and checked the manuscript and edited accordingly for consistency. |
| It would be good if you could provide your questionnaire and interview guide as additional files. This would help readers make sense of why there were clusters with missing data in your framework. Eg. why did you have nothing on accountability/transparency? As you point out, it is surprising. | Thank you for this suggestion. We agree this would be beneficial to readers and have provided the topic guide as a supplementary file. Access to the questionnaire data can be requested by emailing [data-bris@bristol.ac.uk](mailto:data-bris@bristol.ac.uk) (see data access response for full details). |
| Lines 189-195 It’s great that you describe this framework but it made for confusing reading. Possibly because the punctuation wasn’t consistent? I found it hard to identify the main construct and the subconstructs. Perhaps you could number the three main constructs? Table 2 shows them clearly – but that comes later. | Thank you for this suggestion. On reflection we agree that the wording in the text is confusing and think that the table is much clearer for readers. We have therefore removed the narrative description of the framework and signposted readers to the table (Table 1, page 10). We think this makes the framework clearer and hope that you’ll agree. |
| I think it might be more descriptively accessible to refer to ‘researchers’ rather than ‘staff’ when you ascribe quotes. | Thank you, we agree and have changed ‘staff’ to ‘researchers’ in the text. |
| Line 224 -223 and lines 289-299 I think these two sections are dealing with the same issue. Can you find a way to discuss them in only one place? | Thank you for this feedback. We have carefully looked over these sections and the data that informed them. We think that while the two aspects of normative values are similar, they focus on different areas. The first aligns with feelings of empowerment and feeling confident to share views within the group, the second aligns to ethical values and feeling emotionally supported to share personal experiences. We have therefore kept this separate in the results section to map to the model domains. |
| Page 15 – what do staff think about the role of PPI groups in ensuring effectiveness? I find myself wondering if you asked about this. Another reason to supply your questionnaire. | Staff were asked to answer questions designed to address the framework as a whole and using wording that was more reader friendly than the Gradinger framework. To explore effectiveness of the PPI groups on research, staff were asked to reflect on the ‘impact’ of PPI on their research and we have added a quote to the manuscript to reflect this aspect in the staff responses and an addition to Table 1 (Lines 334-335). |
| Lines 345-346 – missing words in this sentence? | Wording added for clarity:  *“One staff member discussed the inclusion of patients in steering group meetings and the impact of having one expert patient.”* – Line 354 |
| Line 419 “different degrees of knowledge” knowledge of what? Clinical issues? Research? | Wording added to clarify as follows:  “…degrees of research methodology and health knowledge (being ‘better at it’) between researchers and group members.” – Lines 428-429 |
| Line 426 – “members did not think that it as their role to make decisions but rather to provide” AS should be WAS | Thank you, our apologies, this has been corrected to read ‘was’. Line – 435 |
| Line 428 - apostrophe - researcher’s should be researchers’ | Thank you, our apologies, this has been corrected. |
| 463-476 – can you blend the two sections here? Maybe use one intro and only one of the quotes as it feels repetitious. | Thank you. We agree that the two quotes reflect similar views. The second quote has been removed to reduce repetition. |
| 548-549 “... or who do not want to be part of a group” How is this changed by online platforms? Can you please clarify. | Wording “who do not want to be part of a group” has been removed. This was included in error, our apologies. |
| It’s great that you engage in a discussion about diversity but I’d like to see a more critical engagement with the concept of representativeness / objectivity / generalisability in PPI, perhaps drawing more broadly on the literature on community consultation. Is generalisability (or objectivity) possible? Certainly not in the statistical sense. So what forms of representation are sought, and with what aims? | Thank you. We agree this is an important point and are pleased to be invited to add further discussion about these concepts. This includes a reflection on the difference between PPI and research, with inclusion in PPI as a way of ensuring that research is designed to meet the needs of people from underserved communities. We have also added some description of community-engagement approaches with Citizens’ Juries as an example that would be seen as a precursor to PPI activities as well as mention of the challenge of disclosing diversity information about small groups to external audiences. We have already discussed in some detail the challenges of disclosure to staff. – Page 25, lines 532-554. |
| Thanks for the opportunity to review this manuscript and good luck with the revisions. I look forward to sharing the final paper with colleagues who are setting up a consumer advisory registry in musculoskeletal research. | Thank you for your helpful feedback and suggestions. We appreciate the time that you’ve taken to review our manuscript and for the expertise that you’ve brought to it. We would be delighted for it to be shared if accepted. |
| **Reviewer 2 feedback** | |
| This is a well written manuscript, presented in an intelligible way. I believe that this work is important for and provides good advice for researchers who will be using PPI groups. My only observation would be regarding the data. If it is anonymised then it would be nice if it was more freely available, perhaps included as supplementary information. | Thank you for this feedback. We appreciate the time that you’ve taken to review the manuscript. We have provided a response to the question about data availability in the editor feedback section above. |
