# Supplementary material for: Value, transparency, and inclusion: A values-based study of patient involvement in musculoskeletal research
Source: PLoS One. 2021 Dec 1;16(12):e0260617. doi: 10.1371/journal.pone.0260617 (PMC8635367; doi:10.1371/journal.pone.0260617)
Supplement: Attachment — Submitted filename: Response to reviewers.docx [file pone.0260617.s003.docx]

PLOS ONE Editorial Team

Carlyle House

Carlyle Road

Cambridge

CB4 3DN

1^st^ September 2021

Dear Sara Rubinelli and Reviewers,

Thank you for taking time to review our manuscript. We very much appreciate the constructive feedback. We have responded to each point raised in the table below. We hope these changes meet with your approval and we look forward to your response.

Yours sincerely,

Katie Whale

| **Feedback** | **Response / action taken** |
| --- | --- |
| **Reviewer 2 feedback** | |
| Lines 96-98 “In assessing the impact of PPI, it has been suggested that understanding theoretical aspects and practical application of patient involvement work provides a fuller picture of how involvement functions” I’m not sure what this sentence means, please can you clarify. | Thank you. We have amended this sentence for clarity.  *“In assessing the impact of PPI, it has been suggested that understanding both the theoretical aspects of PPI and the practical application of patient involvement work provides a fuller picture of how involvement functions.”* |
| Lines 99-100 "...one way to achieve this, enabling scope to review the value of PPI and to identify any areas for further development” Are there words missing from this sentence as it doesn't really make sense? | Thank you. We have added the missing word.  *“Using a theoretical framework to underpin reporting of PPI is one way to achieve this, enabling the scope to review the value of PPI and to identify any areas for further development.”* |
| Lines 101-102 “...identified the existence of a ‘plethora’ of frameworks, tools, and guidelines [? that] exist to support, evaluate, or report PPI” Are there words missing from this sentence? | Thank you. We have added the missing word.  *“A recent systematic review by Greenhalgh and colleagues identified the existence of a ‘plethora’ of frameworks, tools, and guidelines that exist to support, evaluate, or report PPI”* |
